# Face Recognition Deficits in a Patient With Alzheimer's Disease: Amnesia or Agnosia? The Importance of Electrophysiological Markers for Differential Diagnosis

**DOI:** 10.3389/fnagi.2020.580609

**Published:** 2020-12-21

**Authors:** Chiara Mazzi, Gloria Massironi, Javier Sanchez-Lopez, Laura De Togni, Silvia Savazzi

**Affiliations:** ^1^Perception and Awareness (PandA) Lab, University of Verona, Verona, Italy; ^2^Department of Neuroscience, Biomedicine and Movement Sciences, University of Verona, Verona, Italy; ^3^Center for Cognitive Decline and Dementia, ULSS 9 Scaligera, Verona, Italy; ^4^Centro de Investigacion en Ciencias Cognitivas, Universidad Autonoma del Estado de Morelos, Cuernavaca, Mexico

**Keywords:** prosopagnosia, dementia, electrophysiological markers, faces, N170, N250, N400, Alzheimer's disease

## Abstract

Face recognition deficits are frequently reported in Alzheimer's disease (AD) and often attributed to memory impairment. However, it has been hypothesized that failure in identifying familiar people could also be due to deficits in higher-level perceptual processes, since there is evidence showing a reduced inversion effect for faces but not for cars in AD. To address the involvement of these higher processes, we investigated event-related potential (ERP) neural correlates of faces in a patient with AD showing a face recognition deficit. Eight healthy participants were tested as a control group. Participants performed different tasks following the stimulus presentation. In experiment 1, they should indicate whether the stimulus was either a face or a house or a scrambled image. In experiments 2 and 3, they should discriminate between upright and inverted faces (in experiment 2, stimuli were faces with neutral or fearful expressions, while in experiment 3, stimuli were famous or unfamiliar faces). Electrophysiological results reveal that the typical face-specific modulation of the N170 component, which is thought to reflect the structural encoding of faces, was not present in patient MCG, despite being affected by the emotional content of the face implicitly processed by MCG. Conversely, the N400 component, which is thought to reflect the recruitment of the memory trace of the face identity, was found to be implicitly modulated in MCG. These results may identify a possible role for gnosic processes in face recognition deficits in AD and suggest the importance of adopting an integrated approach to the AD diagnosis while considering electrophysiological markers.

## Highlights

- The N400, but not the N170, was modulated in a patient with AD.
- Face recognition deficits of the tested patient were found to have a gnosic nature.
- Electrophysiology can reveal the etiology of neuropsychological deficits in AD.

## Introduction

Alzheimer's disease (AD) is a chronic neurodegenerative disease characterized at a neural level by neuronal atrophy and the presence of amyloid-β plaques and neurofibrillary tangles (Duyckaerts et al., 2009; Jack et al., 2018). The neuropsychological profile of patients with AD is characterized by a variety of problems, such as recalling recent events, language problems, and disorientation. Cognitive functioning declines over time as the disease progresses. Although the first symptoms of AD vary from patient to patient, typically, one of the first signs of the disease referred by the caregivers relates to memory problems. However, other disorders are reported by the caregivers, such as attentional dysfunctions, apraxia, and/or psychiatric and behavioral disturbances (Weintraub et al., 2012). The worsening of cognitive domains and the extension of the impairment to other cognitive functions are the typical patterns of the evolution of AD. At a mild-to-moderate stage of AD, a highly emotionally compelling problem referred by the relatives relates to visuospatial and perceptual problems, specifically the recognition of familiar faces (Greene and Hodges, 1996; Hodges and Greene, 1998). Patients with AD, indeed, are incapable of recognizing known people, including their own relatives, and eventually themselves reflected in a mirror. Problems with recognizing familiar faces have typically been attributed to memory problems, as the progression of the disease worsens (e.g., Becker et al., 1995; Hodges, 2006), impacting on more consolidated memories, such as the memory of familiar people. This is a logical conclusion as recognition implies preserved memory traces. However, a recent study (Lavallée et al., 2016) has shown that face recognition problems in AD may have a different etiology. In their study, 25 mild-stage patients with AD underwent a perceptual task in which they were requested to match simultaneously presented unfamiliar faces (and cars as control stimuli), and stimuli could be either upright or reversed. The rationale of using inverted faces stands in the well-known face inversion effect (Yin, 1969), i.e., an impaired performance in recognizing inverted unfamiliar faces. This effect is thought to reflect different perceptual processing of faces in the upright and inverted orientation (Rossion and Gauthier, 2002), and it is assumed that the larger the effect, the more preserved the ability to process configurational aspects of individual faces. The authors reported that patients with AD enrolled in their study showed a reduced face inversion effect for faces (but not for cars), both in terms of accuracy and the speed of response. Moreover, the performance of patients with AD was not only impaired with inverted faces but also with upright faces, and they suggested that there might be a specific impairment at building a coherent perceptual representation of individual faces in AD.

These results suggest that patients with AD can fail to recognize familiar faces not only because of memory problems but also because of specific deficits in high-level visual processing, thus more properly referable as prosopagnosia (selective agnosia for faces). To further investigate this possibility, we took advantage of electrophysiology and, specifically, event-related potentials (ERPs) to provide reliable neural markers capable of characterizing the neuropsychological profile of cognitive deficits (Rossini et al., 2020). As regards face processing, three main components have been found to correlate with face processing: the N170 (Bötzel et al., 1995; Bentin et al., 1996), the N250 (Schweinberger et al., 1995, 2004), and the N400 components (Bentin and Deouell, 2000; Olivares et al., 2015; Taylor et al., 2016). Although the evidence is somewhat controversial, some studies have also suggested that the P100 may be sensitive to face processing, reflecting either a holistic face perception or differential low-level visual features between faces and other complex visual stimuli (Itier and Taylor, 2004; Rossion and Jacques, 2008).

The N170 component is the earliest and most widely studied negative face-sensitive ERP component. It is a right-lateralized component detected at occipitotemporal electrodes between 140 and 200 ms after the stimulus presentation and is typically larger for faces than for other objects. Importantly, the N170 component is modulated by the orientation of the face (face inversion effect): It is delayed and enhanced for inverted as compared to upright faces (Bentin et al., 1996; Eimer, 2000; Rossion et al., 2000; Sagiv and Bentin, 2001). Moreover, the N170 component is also modulated when the same face is repeated one after another (the identity-dependent adaptation effect, Caharel et al., 2009). Finally, the N170 component has also been shown to be sensitive to the facial expression, with fear eliciting the largest effect as compared to the other emotions (Turano et al., 2017). Taken together, these pieces of evidence suggest that the N170 component represents an electrophysiological marker for the perceptual structural encoding and configurational processing of individual faces.

Unlike N170, the N250 and N400 components are modulated by personal familiarity (Bentin and Deouell, 2000; Webb et al., 2010; Olivares et al., 2015; Taylor et al., 2016). Therefore, they are both considered as indexes of face identification processing, albeit underpinning different aspects. In particular, the N250 component represents the earliest electrophysiological correlate underlying the face recognition process (Pierce et al., 2011; Huang et al., 2017; Wuttke and Schweinberger, 2019). Typically reported at occipitotemporal electrodes with a larger amplitude for famous faces, the N250 component has also been described in the frontal regions at the same latency, though characterized by the opposite pattern, i.e., enhanced negativity for unfamiliar faces compared to famous faces (e.g., Cheng and Pai, 2010; Olivares et al., 2015). Previous literature has associated this component with access to the face perceptual representations stored in visual memory, independent of semantic knowledge (Pierce et al., 2011; Eimer et al., 2012; Schweinberger and Neumann, 2016). This is also because the N250 is modulated by repeated presentation of faces in an experimental context (Schweinberger et al., 2002; Neumann and Schweinberger, 2008; Schweinberger and Neumann, 2016).

By contrast, the N400 is a negative component peaking approximately at 400 ms after the stimulus onset, which is characterized by a centroparietal distribution (Bentin and Deouell, 2000; Eimer, 2000; Olivares et al., 2015). The existing evidence indicates that the N400 component would relate to post-perceptual representation of familiar faces, hence reflecting a purely semantic stage of processing (Bentin and Deouell, 2000; Jemel et al., 2010; Olivares et al., 2015). This hypothesis seems to be in keeping with the enhanced negativity associated with famous faces if compared to unfamiliar faces.

In three different electroencephalogram (EEG) experiments, we assessed the presence and modulation of the three previously described ERP components known to be relevant for face processing. We, thus, tested a patient with AD exhibiting problems in recognizing familiar faces with the aim of defining whether her face processing problems are of mnestic or gnosic nature. Critically, this approach may represent a first step toward evaluating the suitability of electrophysiological markers for differential diagnosis and devising innovative rehabilitation protocols of visuoperceptual deficits in patients with AD.

## The General Method

### Participants

**Figure 1:**
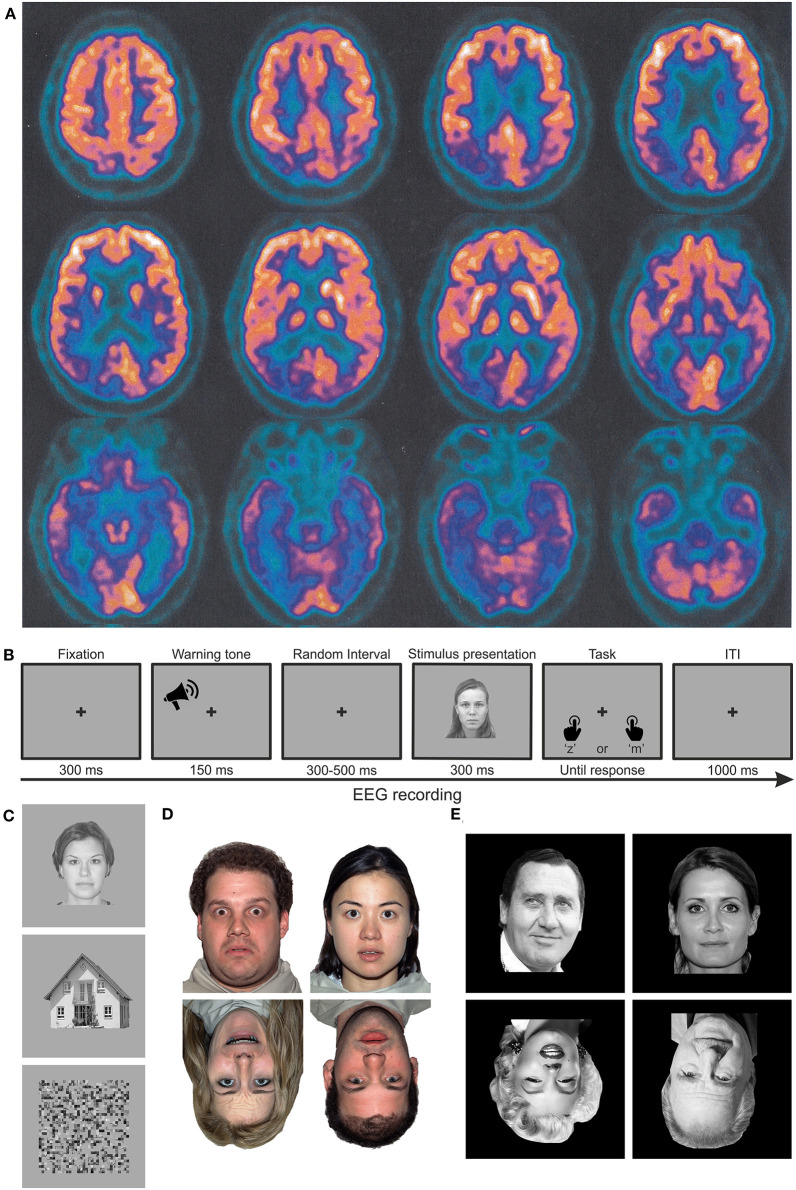
Material and methods. **(A)** Axial PET images of patient MCG at the time of diagnosis, highlighting significant hypometabolism in the bilateral inferior temporal poles and in the occipito-parieto-temporal cortex, lateralized to the right hemisphere. Images are shown according to the radiological convention (right is left and *vice versa*). **(B)** Trial structure of the performed discrimination tasks. In experiment 1, participants had to discriminate whether the stimulus was meaningful or not. In experiments 2 and 3, participants had to discriminate whether the stimulus was upright or inverted. **(C–E)** Examples of stimuli employed in experiments 1 (faces, houses, and scrambled images), 2 (upright and inverted faces with neutral or fearful expressions, respectively), and 3 (upright and inverted faces for famous or unfamiliar faces).

Patient MCG is a 67-year-old right-handed woman with 5 years of education admitted at the Center for Cognitive Decline and Dementia, ULSS 9 Scaligera, Verona, for neurological and neuropsychological assessment. At the time of admittance, MCG is retired but she still helps in the bakery owned by her family. She appears vigilant, collaborating, oriented in both space and time, and conscious of her cognitive status, mainly complaining about recognition problems of familiar faces, e.g., known customers of the bakery. Instrumental and neuropsychological tests (see dedicated section below) are requested. Since she could not be admitted to the MRI room because of the presence of a pacemaker, she was administered with a CT scan and a PET. The CT scan did not show any clear sign of tissue hypodensity or atrophy (image not available). In contrast, PET data evidenced hypometabolism of the inferior temporal cortex bilaterally and the occipitotemporal and parietotemporal cortices in the right hemisphere (Figure 1A). Moreover, the cerebrospinal fluid (CSF) test evidenced a biomarker (amyloid-β and Tau) profile compatible with AD.

Based upon the previous literature (e.g., Prieto, 2011), eight healthy right-handed female participants (age range: 63–70) with no history of neurological or psychiatric disorders were tested as a control group. The patient and healthy participants signed the informed consent prior to participating in the study and were free to withdraw at any time. The study was approved by the local ethics committee and conducted in accordance with the 2013 Declaration of Helsinki.

### Neuropsychological Testing

Patient MCG underwent a comprehensive neuropsychological battery (Table 1) for the assessment of cognitive functions. Each test was administered and scored using standard procedures and instructions. The entire neuropsychological assessment lasted for about 50 min. General cognitive impairment was assessed by means of the Mini Mental State Examination (MMSE, Magni et al., 1996), whereas specific cognitive functions were assessed by means of *ad hoc* tests. The Trial Making Test (Giovagnoli et al., 1996) was used to assess attentional functions. Visuospatial and praxis abilities were assessed by means of the version of the Clock Drawing Test (Sunderland et al., 1989) specifically standardized for AD patients. Three tests were used to assess language functions: a short version of the Token Test (Mondini et al., 2011) to assess language comprehension and two tests of verbal fluency to assess phonological (Carlesimo et al., 1996) and semantic (Novelli et al., 1986) access. The Rey Auditory Verbal Learning Test (Rey, 1958; Carlesimo et al., 1996) was used to assess short-term and long-term memory impairment. Logical reasoning was assessed by means of the abstraction test (Mondini et al., 2011). Two tests were used to assess constructional apraxia (Carlesimo et al., 1996) and ideational and ideomotor apraxia (Mondini et al., 2011). Finally, depressive symptoms were assessed by means of the Geriatric Depression Scale (GDS, Sheikh and Yesavage, 1986), showing a slight mood deflection.

**Table 1:** Neuropsychological assessment.

**Function**	**Test/sub-component**	r.s.^*^	c.s.^*^	e.s.^*^	**Evaluation**
General cognitive abilities	MMSE	24/30	22.9/30	–	Mild deficit
Verbal learning	Immediate recall	39/75	45.1/75	4	In the norm
(Rey 15-words)	Delayed recall	7/15	8.8/15	4	In the norm
	Correct recognitions	11/15	–	–	–
	Missed recognitions	4	–	–	**Impaired**
	False recognitions	0	–	–	–
Attention	Trail making test A	420	396	0	**Impaired**
Language	Phonological fluency	13	20.9	1	At the lower limit of the norm
	Semantic fluency	24	32	2	In the norm
	Language comprehension (Token test)	5/5	Cut off=5	4	In the norm
Visuospatial and praxis abilities	Clock drawing test	6/10	–	–	At the lower limit of the norm
Logical reasoning	Abstraction test	4/6	Cut off=3	–	In the norm
Praxis	Constructional apraxia	3/12	4.1/12	0	**Impaired**
	Ideational and ideomotor apraxia	5/6	Cut off=6	–	**Impaired**
Depression	Geriatric depression scale	7/15	Cut off>5	–	Deflection

* *r.s., raw scores; c.s., correct score; e.s., equivalent score. Bold characters were just used to highlight where the performance was impaired*.

As assessed during the clinical colloquium and as reported in Table 1, MCG appeared to have difficulties in some cognitive functions, including having vocabulary in the lower limit of the norm for phonemic cue and in the norm for semantic cue. Whereas, MCG appeared to have fluency in spontaneous speech, correct in both form and content, a normal limit in verbal understanding, and a good autobiographical memory. The ability to learn verbal stimuli was in the norm, with a good ability to recover the information previously stored at a deferred recall. At the incidental memory test of the MMSE, all three words were recovered. Visual processing, number recognition, knowledge, and reproduction of numerical sequences with motor slowdown, tested with the Trial Making Test, were impaired. However, the ability to perform mental backward tasks was partially preserved. The planning of the clock drawing was sufficient: The sequence of numbers was correct but with slight inaccuracies in the spatial arrangement; the position of the hands of the clock on the requested time was incorrect, showing a slight difficulty in mentally representing a clock. Instead, the logical-deductive capacities were in the norm. Finally, the ability to copy geometric figures (constructional apraxia) was seriously compromised while the ideational and ideomotor praxis abilities were impaired.

During the neuropsychological testing and as reported by her daughter, MCG experienced difficulties in recognizing familiar faces. A preliminary assessment of face recognition abilities was performed by showing MCG black-and-white 10 × 20 cm pictures (faces only) of famous people (10 males and 10 females; two images of the same individual, one at an young age and one at an older age, were administered). Faces were shown one at a time, and MCG was asked to state the name of the person shown in the picture and provide any other information she could recollect of that person. She could not identify any of the famous faces, with the exception of the image of an aged Silvio Berlusconi (former Italian Prime Minister), as previously described by Mondini and Semenza (2006), despite recognizing the images as a face and reporting details of the faces (e.g., the beard of *Padre* Pio). She also could not identify the emotions expressed by the faces. In a second test, the same images were shown three at a time, two versions of the same person and one of a different person. The task of MCG was to find the image not belonging to the same person. In this case, she could not perform the task, as she was unable to match the two images belonging to the same identity.

Moreover, to ascertain the specificity of the agnosia deficit, an object recognition test was programed for the second visit. One week after the general neuropsychological testing, MCG was tested with a standardized set of 260 pictures of objects (Snodgrass and Vanderwart, 1980) comprising both living and non-living objects. This testing lasted for about 1½ h. MCG committed a total of 41 identification errors (15.8%), with more errors for the non-living (9.6%) than the living (6.2%) objects. In all cases of identification errors, she could, nonetheless, recognize the semantic category of the objects, as the reported name was of an object of the same category and with similar physical characteristics most of the times. These results show mild difficulties in recognizing objects, non-comparable to the pervasive deficit in recognizing faces.

### Experimental Design, Apparatus, and Stimuli

In all the experiments, participants were seated in a comfortable chair in front of a monitor in a dimly lit room. An adjustable chin and forehead rest was used to minimize head movements, ensuring that the distance between the participant and the monitor remained constant at 57 cm. Visual stimuli were presented using E-prime2 (Psychology Software Tools) *via* a 17-in IBM G96 CRT refreshing at 85 Hz (resolution 1,280 × 1,024 pixels). Online monitoring of eye movements was performed by an infrared camera in order to verify the maintenance of fixation during the stimulus presentation. Different stimuli were tested in three different experiments. The three experiments were run on different days for patient MCG and on the same day for the healthy controls.

Figure 1B illustrates the experimental procedure. Each trial started with the appearance of a central fixation cross for 300 ms, which lasted throughout the entire trial. Stimulus presentation was preceded by a 1,000 Hz warning acoustic tone lasting 150 ms. To avoid any expectation, the interval between the warning tone and the stimulus onset was randomized between a 300–600 ms time window. After a 1,000 ms pause, a prompt response was given, asking the participant to perform a discrimination task by pressing two different buttons of the keyboard.

Stimuli presented in all experiments subtended 12° × 12° of the visual angle and was presented for 300 ms. Stimuli were presented centrally in the visual field, and different types of stimuli were presented during the three experiments (see below). After a 1,000 ms intertrial interval, the next trial was presented.

### EEG Recording, Preprocessing, and Event-Related Brain Potential Analysis

Electroencephalogram (EEG) signal was continuously recorded with the BrainAmp system (Brain Products GmbH, Munich, Germany—BrainVision Recorder) using a Fast'n Easy cap with 59 Ag/AgCl pellet pin electrodes (EasyCap GmbH, Herrsching, Germany) placed according to the 10–10 International System. Four additional electrodes were used for monitoring blinks and eye movements. Horizontal and vertical eye movements were detected with electrodes placed at the left and right canthi and above and below the right eye, respectively. Other two extra electrodes served as a ground reference (AFz) and as an online reference (right mastoid, RM). Electrode impedances were kept below 10 kΩ. The digitization rate was 1,000 Hz with a time constant of 10 s as low cut-off and a high cut-off of 250 Hz.

The continuous EEG signal was processed off-line using EEGLAB (v14_1, Swartz Center for Computational Neuroscience, University of California at San Diego, Delorme and Makeig, 2004). Data were first down-sampled to 250 Hz and high-pass filtered at 1 Hz. Scalp channels were then offline re-referenced to the average of all electrodes prior to using the CleanLine EEGLAB plugin (Mullen, 2012) in order to reduce noise in the power line (50 Hz and its harmonics) by means of the adaptive multitaper regression. Independent component analysis (ICA) using the extended InfoMax algorithm (Bell and Sejnowski, 1995) was performed on the segmented data (from −1,000 to 1,000 ms with respect to the stimulus onset). The independent components identified as artifactual (e.g., blinks, eye movements, or muscle activity) were removed according to visual inspection and, subsequently, a low-pass filter at 40 Hz was applied. The epoch window was then shortened starting from 300 ms before the stimulus onset to 800 ms after the stimulus presentation and, thereafter, the baseline was corrected using the prestimulus interval. Artifact rejection was performed manually to exclude those segments contaminated by residual isolated artifacts. Finally, the retained data were averaged across experimental conditions for each electrode and for each participant.

### Statistical Analysis

#### Behavioral Data

Data were processed using MATLAB 2019a and analyzed with IBM SPSS Statistics for Windows, version 22. Either no-response or incorrect trials were excluded from behavioral analyses. Furthermore, trials with response times (RTs) exceeding ±3SDs from the mean in each experimental condition were labeled as outliers and removed from the dataset as well. Repeated measures ANOVAs were performed on both accuracy and RTs of the control group in order to detect possible differences in performance among the different conditions. As regards patient data, the statistical difference between conditions in both accuracy and RTs was assessed by means of a non-parametric bootstrap procedure using 1,000 replications (α = 0.05). To assess whether the performance of MCG was significantly higher than the chance level (50%) within each condition, binomial tests were performed. A series of Crawford's *t*-test for a single case analysis (Crawford and Howell, 1998; Crawford and Garthwaite, 2002; Crawford et al., 2010) was performed for the comparison of the performance between patient MCG and the control group (accuracy and RTs).

#### EEG Data

The time windows and the electrodes chosen for the analyses were selected according to both the previous literature and the visual inspection of the waveforms. Indeed, only the electrode showing the highest amplitude within the typical time window and the scalp topography of the component of interest was entered into the statistical analysis. In particular, in all the three experiments, the N170 component has been assessed in electrodes P8 and T8 for the control group and the patient MCG, respectively. Electrode F2 has been considered for the N250 component, while electrodes P2 for healthy controls and P6 for patient MCG were the most prominent sites for the N400 component.

Grand-average ERPs of the selected sites were submitted to the repeated measures ANOVAs implemented in the EEGLAB study. The main effects were computed using parametric statistical routines with a statistical threshold of 0.05. As regards patient data, statistical differences among conditions were evaluated using permutation-based statistics (α = 0.05, 1,000 replications), which was implemented in the EEGLAB.

## Experiment 1—Face Processing

The purpose of this experiment is to ascertain whether it is possible to find markers of face processing in the patient. The N170 component will be investigated. In healthy participants, as reported in the literature, we expect to find a larger N170 for faces than for houses and scrambled images. If the face recognition deficit of patient MCG is due to memory problems, we should expect no difference in the results between healthy participants and the patient. Conversely, if her face recognition deficit is due to gnosic problems, thus reflecting an inability to create a coherent percept and to process perceptual information up to the level of the meaning of the percept itself, we should not expect to find a larger N170 for faces than for houses and scramble images.

### Stimuli and Design

Stimuli (Figure 1C) were of three categories: (male and female) faces, houses, and scrambled images. Face and house stimuli were taken from the NimStim database (Tottenham et al., 2009). Scrambled stimuli were created by scrambling the images of faces and houses by means of a custom-made MATLAB script. The experiment included 72 different faces, 72 different houses, and 144 different scrambled images generated from the same meaningful images. Each stimulus identity was repeated twice. Stimuli were grayscale images with a background luminance of 8.56 cd/m^2^.

The experiment was divided into 24 blocks of 24 trials each (six faces, six houses, and 12 scrambled images), thus yielding a total of 576 trials. The number of total trials was reduced to 480 for healthy participants, subdivided into 20 blocks of 24 trials each. The order of the trials was fully randomized within each participant.

The participants had to report whether the stimulus was meaningful (i.e., a face or a house) or not (i.e., a scrambled image) by pressing two different keys (“m” and “z” keys, respectively) on the keyboard.

EEG averaging was carried out separately for the three different conditions: faces, houses, and scrambled images.

### Results and Discussion

**Figure 2:**
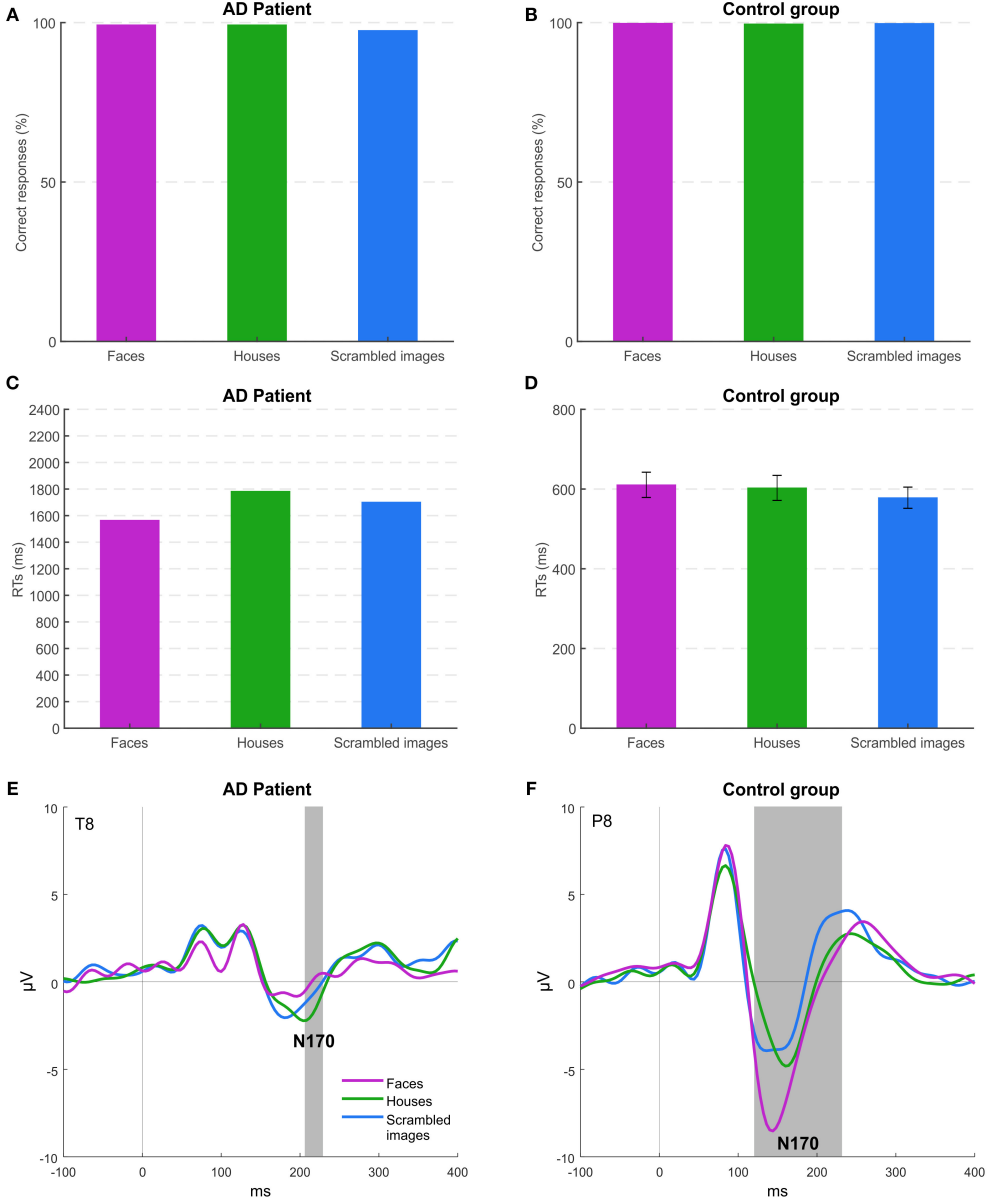
Experiment 1: behavioral and EEG results. **(A,B)** Mean accuracies across the different stimuli for patient MCG and the control group. **(C,D)** Mean response times (RTs) of correct trials across the different stimuli for patient MCG and the control group (error bars represent SEM). **(E,F)** Grand-average event-related potentials (ERPs) for each condition. Gray areas indicate significant time windows for the N170 component (category effect), respectively, for electrode T8 (patient MCG), and electrode P8 (control group).

Figure 2 shows accuracy and RTs of MCG and controls for all the stimulus categories. The high level of accuracy across conditions (overall >99% for controls and 98% for the patient) indicates that both controls and, more importantly, patient MCG could discriminate the category of the stimuli, being able to distinguish between meaningful (faces and houses) and meaningless (scrambled) objects. Neither controls [*F*_(2, 14)_ = 0.542; *p* = 0.593] nor the patient (*p* > 0.05) highlighted differences in accuracy among conditions. Face and house accuracy levels did not differ significantly for patient MCG compared to controls [faces *t*_(7)_ = −1.179; p = 0.277; houses *t*_(7)_ = −0.404; *p* = 0.698], while the accuracy for scrambled stimuli was lower in patient MCG than in controls [*t*_(7)_ = −4.148; *p* < 0.01]. The RTs of the patient were slower than those of the control group for all the conditions considered [faces *t*_(7)_ = 5.025; *p* < 0.01; houses *t*_(7)_ = 6.250; *p* < 0.001; scrambled images *t*_(7)_ = 7.052; *p* < 0.001]. Moreover, the patient reacted quicker (*p* < 0.05) for faces (1,565 ms) than for houses (1,783 ms) and scrambled images (1,701 ms), while healthy controls RTs did not highlight any differences [*F*_(2, 14)_ = 1.217; *p* = 0.326] (611, 603, and 579 ms, respectively). These results are not unexpected, even in patient MCG as she has demonstrated to have, at a behavioral level, the ability to categorize a face as a face and a house as a house during the neuropsychological assessment. Her deficit, indeed, was not related to the recognition of a face as a configuration of elements, but to the attribution of a specific personal identity to a familiar face.

Event-Related Potential (ERP) analysis, thus, becomes essential to understand whether this recognition deficit in patient MCG is related to either the perceptual or the memory component of information processing eventually resulting to face identity recognition. In controls (Figure 2F), as expected, the amplitude of the N170 component was higher for faces than for the other stimuli (electrode P8 from 120 to 236 ms, *p* < 0.05). In MCG (Figure 2E), instead, we found a strongly reduced N170 component for all the stimuli presented with an even significant smaller amplitude for faces (electrode T8 from 208 to 228 ms, *p* < 0.05) than for houses and scrambled images. These results suggest that MCG recognition problem is related to difficulties in the structural encoding of individual faces (i.e., not for faces in general). Indeed, the N170 component, although not being affected by long-term familiarity for a face, it is modulated by systematic repetition of individual unfamiliar faces, even regardless of their viewpoint (Jemel et al., 2005; Caharel et al., 2009), thus indicating that the N170 component is an electrophysiological marker encoding the specific configuration of elements of the face belonging to specific individuals (Eimer, 2011).

## Experiment 2—Effects of Face Inversion and Emotion

The purpose of this experiment is to ascertain whether it is possible to elicit an N170 component in patient MCG by taking advantage of the “inversion effect” (Bentin et al., 1996; Rossion et al., 1999, 2000) and by using faces with emotional expressions. Indeed, as previously stated, the N170 component in healthy participants has been found to be enhanced for inverted (upside-down) faces. Moreover, despite the patient cannot consciously report the emotional content of faces, we cannot exclude implicit processing of emotions, especially of fear (Morris et al., 1999, 2001; Vuilleumier et al., 2002; Blau et al., 2007; Cecere et al., 2014), thanks to the recruitment of both subcortical and cortical structures devoted to the processing of threatening stimuli such as part of the limbic system (e.g., amygdala) and the superior temporal sulcus (Öhman, 2005; Miyahara et al., 2013; Tseng et al., 2014). Based on the results of experiment 1, we thus expect to find a small, if present, N170 component for upright and neutral faces. Moreover, we investigate the presence of an “inversion effect” and we test whether implicit processing of emotion expressions can emerge, which is expected to elicit an enhancement of the N170 component for fearful faces. If face recognition problems of MCG are of gnosic nature, we should not find a reliable enhancement of the N170 with upside-down faces. With respect to the emotional effect, instead, we cannot formulate a precise prediction as the implicit processing is still possible in neurological patients but not predictable.

### Stimuli and Design

In experiment 2, only (male and female) faces were presented. Stimuli (Figure 1D) were selected from the NimStim database (Tottenham et al., 2009). The experiment included 36 different faces with two emotional expressions (neutral | fearful). Each face was repeated four times. Stimuli were colored images with a background luminance of 61.45 cd/m^2^.

The experiment was divided into twenty-four blocks of 24 trials each (six neutral upright faces, six neutral inverted faces, six fearful upright faces, six fearful inverted faces), thus yielding a total of 576 trials. The number of total trials was reduced to 480 for healthy participants, subdivided into 20 blocks of 24 trials each. The order of the trials was fully randomized within each participant.

The participants had to report whether the stimulus was upright or inverted by pressing two different keys (“m” and “z” keys, respectively) on the keyboard. EEG averaging was carried out separately for the four different stimulus categories.

### Results and Discussion

**Figure 3:**
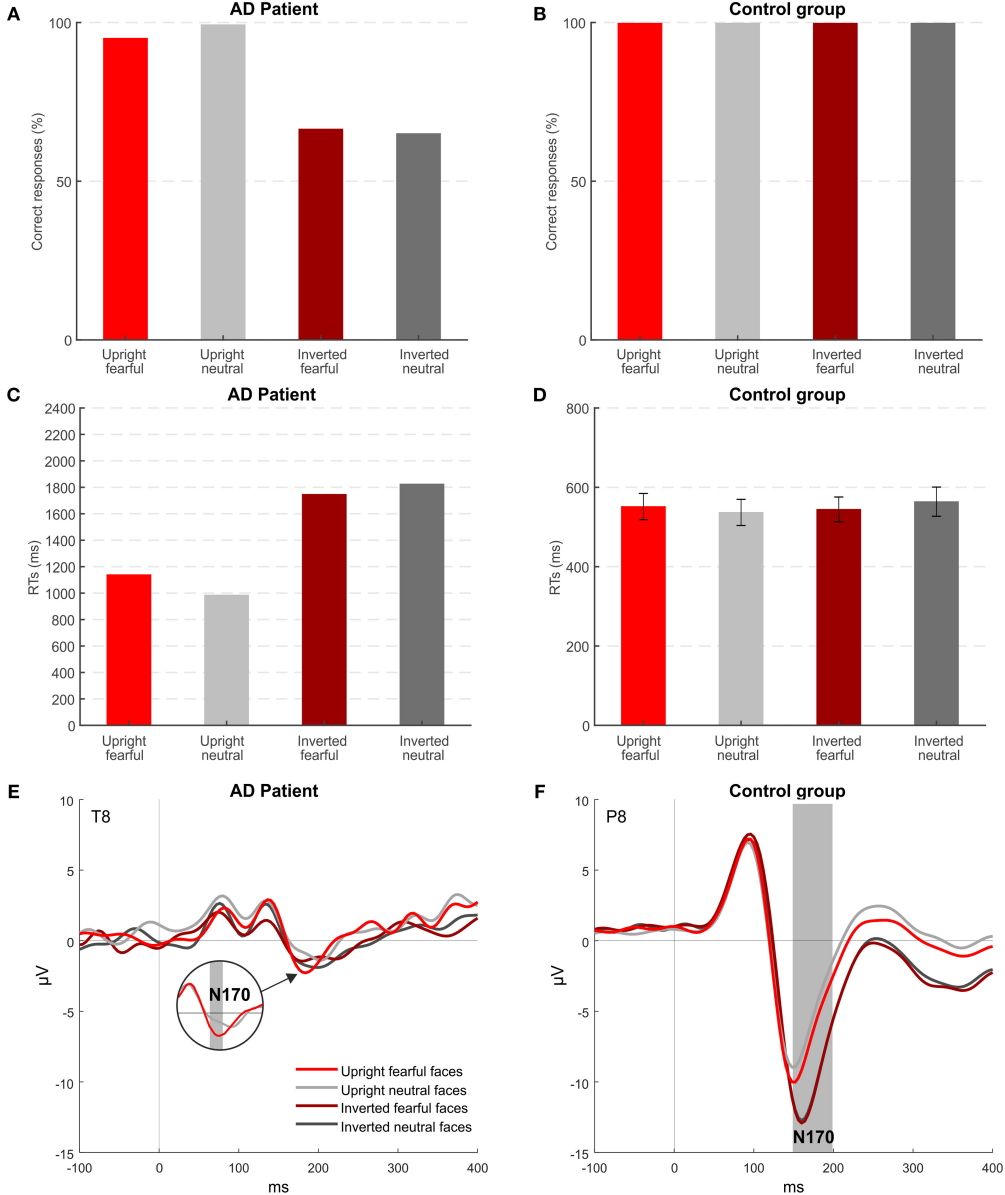
Experiment 2: behavioral and EEG results. **(A,B)** Mean accuracies across the different stimuli for patient MCG and the control group. **(C,D)** Mean RTs of correct trials across the different stimuli for patient MCG and the control group (error bars represent SEM). **(E,F)** Grand-average ERPs for each condition. Gray areas indicate significant time windows for the N170 component (inversion effect and emotional effect) for electrode T8 (patient MCG) and electrode P8 (control group).

Figure 3 shows accuracy and RTs of patient MCG and controls for the four conditions of stimulation. Healthy controls had an accuracy at the ceiling level for all the conditions {overall >99.5%; orientation [*F*_(1, 7)_ = 0.001; *p* = 0.974], emotion [*F*_(1, 7)_ = 0.001; *p* = 0.985], orientation × emotion [*F*_(1, 7)_ = 0.001; *p* = 0.992]}. Instead, MCG was more accurate for upright faces (97%), while her accuracy lowered for inverted faces (66%), despite still being higher than the chance level (all *ps* < 0.001). Accuracy levels differed significantly for patient MCG compared to the control group [upright fearful faces *t*_(7)_ = −11.314; *p* < 0.001; inverted fearful faces *t*_(7)_ = −78.725; *p* < 0.001; inverted neutral faces *t*_(7)_ = −82.024; *p* < 0.001], except for upright neutral faces [*t*_(7)_ = −1.179; p = 0.277]. Looking at RTs, patient MCG was slower than the control group in all the conditions considered [upright fearful faces *t*_(7)_ = 2.960; *p* < 0.05; upright neutral faces *t*_(7)_ = 2.271; *p* < 0.05; inverted fearful faces *t*_(7)_ = 6.387; *p* < 0.001; inverted neutral faces *t*_(7)_ = 5.711; *p* < 0.001]. While not finding any significant differences within the control group {upright fearful faces 552 ms, upright neutral faces 537 ms, inverted fearful faces 548 ms, and inverted neutral faces 565 ms; orientation [*F*_(1, 7)_ = 0.793; *p* = 0.40], emotion [*F*_(1, 7)_ = 0.013; *p* = 0.912], orientation × emotion [*F*_(1, 7)_ = 2.758; *p* = 0.141]}, the patient was quicker when the stimuli were presented upright (upright faces 1,062 ms, inverted faces 1,785 ms, *p* < 0.05), regardless of the expressed emotion. These results indicate that MCG seems to have some difficulties in detecting a face as upside-down.

Event-related potential (ERP) results show that, in healthy participants (Figure 3F), the N170 component was modulated by both the orientation of faces and the emotional content of the upright faces (electrode P8, time window from 152 to 200, *p* < 0.05). As expected, based on the literature, the N170 component was enhanced when upside-down (Bentin et al., 1996) and fearful faces (Turano et al., 2017) were presented. Patient MCG (Figure 3E) did not show any inversion effect. Indeed, her N170 was overall attenuated with no significant difference between upright and inverted faces. However, MCG showed an effect of the emotional content (Figure 3E): The N170 component elicited by upright fearful faces was significantly greater than that elicited by upright neutral faces (electrode T8, time window from 176 to 180, *p* < 0.05). Importantly, this effect was implicit, as MCG could not consciously report the emotional content of the face. Indeed, when directly asked to report the emotional content of a fearful face, she reported that the person “had a nice smile,” thus indicating that she can detect the presence of an open mouth showing teeth despite not attributing the correct meaning to this specific configuration. The implicit effect of emotion detection could be mediated by subcortical centers, belonging to the limbic system, known to be the neural correlate of emotion detection, mainly recruited by negative emotions (e.g., Bennett and Hacker, 2005).

## Experiment 3—Effects of Face Inversion and Familiarity

The purpose of this experiment is two-fold: to replicate the lack of the “inversion effect” found in the previous experiment, i.e., the absence of an enhanced N170 component for inverted faces in MCG, and, importantly, to investigate whether it is possible to find neural markers for an implicit (since she could not overtly report the identity of the faces) “familiarity effect” by examining the components typically modulated by familiarity (i.e., N250 and N400, Bentin and Deouell, 2000; Cheng and Pai, 2010; Pierce et al., 2011; Taylor et al., 2016; Huang et al., 2017; Wuttke and Schweinberger, 2019). As previously stated, in line with the literature, an enhancement of the N170 component for inverted faces (“inversion effect”) should be found in healthy participants, and, based on the results of experiment 2, this effect should not be present in patient MCG. Moreover, if patient MCG can process the identity of the face, at least implicitly, we expect to find an effect on the N250 or the N400 components as typically found in healthy participants.

### Stimuli and Design

Also, in experiment 3, only faces were presented. Stimuli (Figure 1E) were male and female faces of famous and unknown people selected from a database used to ascertain familiarity in unpublished experiments on 50 participants. Moreover, the faces of famous people were presented to a relative of MCG (her daughter) to make sure that these faces were familiar to the patient before the illness. The experiment included two photographs (taken from a different perspective) of each of the 36 different presented people (18 males and 18 females), half of them famous and half not. Each photograph was repeated four times. Stimuli were black and white images with a background luminance of 0 cd/m^2^.

The experiment was divided into 24 blocks of 24 trials each (six famous upright faces, six famous inverted faces, six unknown upright faces, and six unknown inverted faces), thus yielding a total of 576 trials. The order of the trials was fully randomized within each participant. The participants had to report whether the stimulus was upright or inverted by pressing two different keys (“m” and “z” keys, respectively) on the keyboard. EEG averaging was carried out separately for the four different stimulus categories.

### Results and Discussion

**Figure 4:**
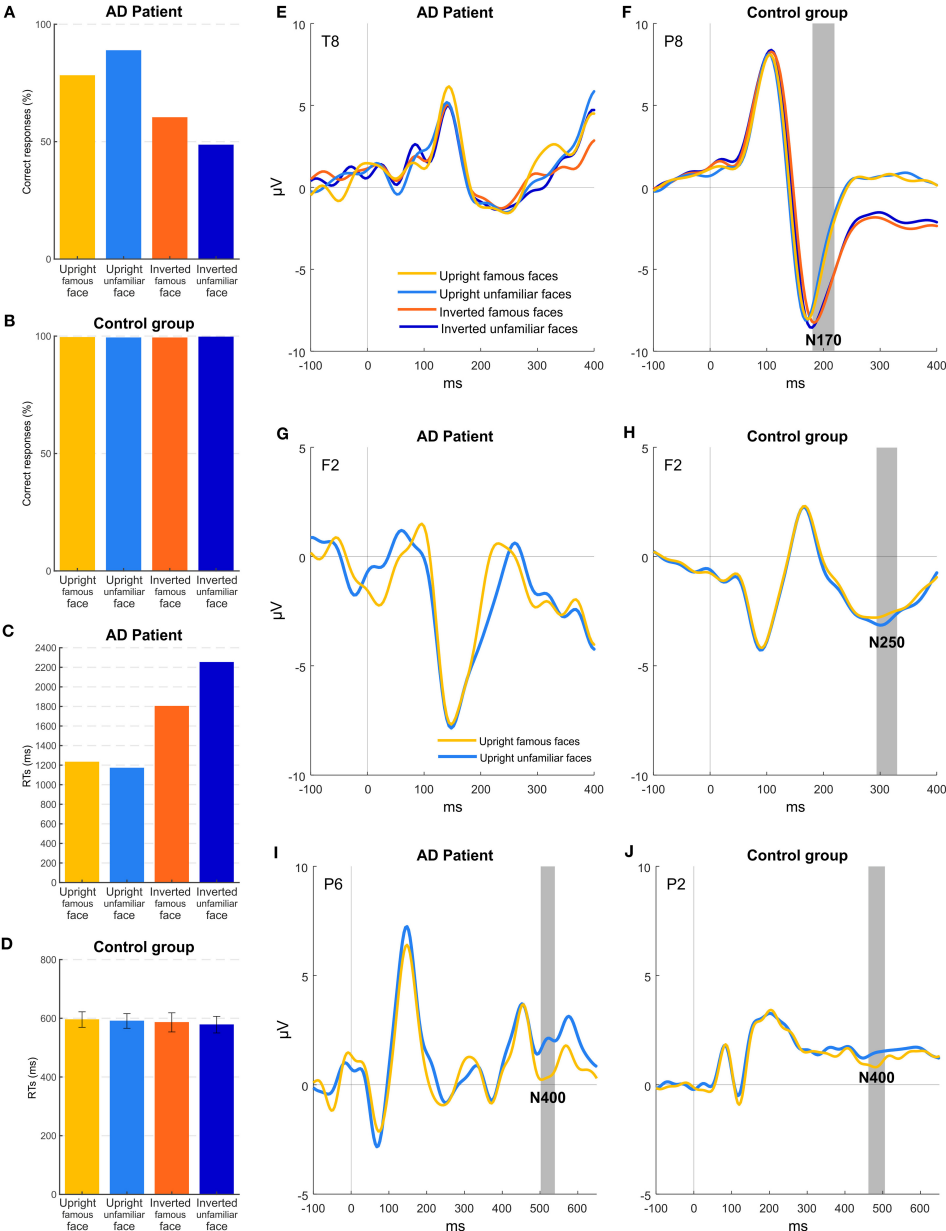
Experiment 3: behavioral and EEG results. **(A,B)** Mean accuracies across the different stimuli for patient MCG and the control group. **(C,D)** Mean RTs of correct trials across the different stimuli for patient MCG and the control group (error bars represent SEM). **(E,F)** Grand-average ERPs for each condition. Gray areas (if present) indicate significant time windows for the N170 component (inversion effect) for electrode T8 (patient MCG) and electrode P8 (control group). **(G,H)** Grand-average ERPs for upright conditions. Gray areas (if present) indicate significant time windows for the N250 component (familiarity effect) for electrode F2 (patient MCG and control group). **(I,J)** Grand-average ERPs for upright conditions. Gray areas indicate significant time windows for the N400 component (familiarity effect) for electrode P6 (patient MCG) and electrode P2 (control group).

Figure 4 shows accuracy and RTs of patient MCG and the control group for the four conditions of stimulation. Healthy controls had an accuracy at the ceiling level for all the conditions {overall >99%; orientation [*F*_(1, 7)_ = 0.576; *p* = 0.473], familiarity [*F*_(1, 7)_ = 0.090; *p* = 0.773], orientation × familiarity [*F*_(1, 7)_ = 5.812; *p* < 0.05]}. Instead, MCG, similar to experiment 2, was more accurate for upright faces (83%), while her accuracy lowered for inverted face (55%), despite still being higher than the chance level (all *ps* < 0.05) for all conditions except for the inverted unfamiliar faces (49%, *p* = 0.934). Accuracy levels differed significantly for patient MCG compared to the control group [upright famous faces *t*_(7)_ = −19.152; *p* < 0.001; upright unfamiliar faces *t*_(7)_ = −10.999; *p* < 0.001; inverted famous faces *t*_(7)_ = −33.427; *p* < 0.001; inverted unfamiliar faces *t*_(7)_ = −96.335; *p* < 0.001]. Looking at the RTs, patient MCG was slower than the control group in all the conditions considered [upright famous faces *t*_(7)_ = 3.933; *p* < 0.01; upright unfamiliar faces *t*_(7)_ = 3.915; *p* < 0.01; inverted famous faces *t*_(7)_ = 6.078; *p* < 0.001; inverted unfamiliar faces *t*_(7)_ = 9.920; *p* < 0.001]. While not finding any significant main effect within the control group {upright famous faces 598 ms, upright unfamiliar faces 597 ms, inverted famous faces 590 ms, and inverted unfamiliar faces 578 ms; orientation [*F*_(1, 7)_ = 0.360; *p* = 0.567], familiarity [*F*_(1, 7)_ = 0.190; *p* = 0.676], orientation × familiarity [*F*_(1, 7)_ = 0.256; *p* = 0.628]}, the patient was quicker when the stimuli were presented upright (upright faces 1,201 ms, inverted faces 2,026 ms, *p* < 0.05), regardless of the familiarity. Replicating the results of experiment 2, MCG, thus, seems to show some difficulties in detecting a face as upside-down.

As in experiment 2, ERP results show that, in healthy participants (Figure 4F), the N170 component was modulated by the orientation of the faces (electrode P8, time window from 188 to 200, *p* < 0.05), with a greater amplitude for upside-down faces. Importantly, healthy participants showed also a significant effect of familiarity (Figure 4H), with a larger frontal N250 component (hereafter called fN250) for upright unfamiliar faces than famous faces (electrode F2, time window from 292 to 328, *p* < 0.05) (see also Cheng and Pai, 2010; Olivares et al., 2015). Remarkably, the reversed effect (i.e., an enhanced amplitude for familiar than for unfamiliar stimuli), though not significant, could be appreciated at posterior temporal sites as well, as consistently reported in the literature (Bentin and Deouell, 2000; Pierce et al., 2011). Indeed, an additional check was performed to exclude that our frontal N250 was due to random noise without any functional meaning. Furthermore, an effect was found considering the N400 component. In particular, the N400 showed (Figure 4J) a greater amplitude in the 504–540 time window for upright famous faces compared to upright unfamiliar faces (electrode P2, *p* < 0.05).

Patient MCG did not show any inversion effect: Replicating the results of experiment 2, the N170 was overall very attenuated as compared to controls, and no significant difference was found between upright and inverted faces (Figure 4E). Importantly, no implicit effect of familiarity was found if considering the N250 component (Figure 4G). Indeed, no differences between famous and unfamiliar faces were observed at any electrodes in the corresponding time window. Conversely, the N400 component (Figure 4I) was significantly modulated as a function of the familiarity of the upright stimuli (electrode P6, time window from 504 to 540, *p* < 0.05).

Taken together, these results corroborate the hypothesis that difficulties of MCG in recognizing faces are of gnosic nature. Interestingly, her difficulties in encoding the structural properties of individual faces (lack of the inversion effect on the N170 component) have not prevented her from implicitly processing the identity of the faces she was presented with. Indeed, despite the lack of modulation of the N250 component, MCG showed a reliable modulation of the N400, in line with the possibility that the face identity was covertly processed. Although the modulation of the N400 together with a lack of a reliable N170 could seem to be counterintuitive, these results can be explained considering the nature of the two components. The N170 is known to correlate with structural properties of a face necessary to be processed to recognize a face (high-level processing of face details). On the other hand, the N400 reflects the matching of a perceived face with the relative semantic representation stored in memory (Eimer, 2000). In principle, this matching could be made at a more abstract level of face representation, i.e., irrespective of its orientation, size, etc. Thus, it might be possible to reach this match even with an impaired face structural encoding, which would render the patient overtly unable to recognize the face but still be capable of implicitly processing it at the level of its memory trace. This result is important as it corroborates the claim that the face recognition problems of MCG might be of gnosic and not of mnestic nature. Previous evidence has already documented covert face recognition in prosopagnosic patients, suggesting an indirect access to those memory traces they cannot use overtly (Bruyer, 1991; De Haan et al., 1991; Avidan and Behrmann, 2008). Although several tasks and psychophysiological measures (e.g., face priming paradigms, forced-choice familiarity task, and skin conductance response) have been employed in order to understand whether overt and covert face recognition mechanisms are subserved by functional distinct pathways, the underlying neural dynamics are still a matter of debate. ERPs have also been used with this aim and different electrophysiological components emerging at least 200 ms after the onset of stimulus have been hypothesized to be responsible for implicit face recognition processing. A pioneering study (Renault et al., 1989) found that the amplitude of the P300 was modulated by familiar and unfamiliar face processing, while another paper (Bobes et al., 2003) reported an effect on the N300. Furthermore, in contrast with our results, the N250, but not the P600, was found to be a marker of covert face recognition in developmental prosopagnosia (Eimer et al., 2012).

## General Conclusions

In the present paper, we tested a patient with AD (and a control group of healthy participants) on her ability to process face stimuli in three different experiments. Importantly, EEG recording was used to find neural markers of face processing, and experimental manipulations were designed to try dissociating the perceptual from the memory component of face processing and recognition. Indeed, the modulation of different ERP components has been found to correlate with different aspects of face processing. The N170 reflects the perceptual component of the generation of a coherent configuration of elements, eventually resulting in identifying a face as a face, whereas the N250 and the N400 reflect the memory component of the association of this specific configuration to the face identity. Specifically, the N250 seems to relate to the interplay between perceptual and memory stages (Pierce et al., 2011; Eimer et al., 2012; Schweinberger and Neumann, 2016), while the N400 seems to account for post-perceptual processing, only related to the memory stage (Bentin and Deouell, 2000; Jemel et al., 2010; Olivares et al., 2015).

Results of patient MCG in experiment 1 showed that, at a behavioral level, she could discriminate between meaningful (faces and houses) and meaningless objects, in line with her ability to recognize a face as a face. In contrast, ERP data have shown that the N170, though being small in absolute terms, was significantly smaller for faces than for houses and scrambled objects, thus accounting for a deficit that could be ascribed to difficulties in encoding the specific configurational and structural elements of faces belonging to specific individuals (Eimer, 2011).

In both experiments 2 and 3, where the patient was asked to discriminate between upright and inverted faces, data showed a relatively preserved ability to recognize face orientation by reacting significantly faster and more accurately when the face stimuli were presented upright. Importantly, contrary to control participants, MCG did not show any inversion effect at the ERP level, i.e., a delayed and enhanced N170 component for inverted faces as compared to upright ones (Bentin et al., 1996; Eimer, 2000; Rossion et al., 2000; Sagiv and Bentin, 2001), thus reinforcing the claim that face recognition problems of MCG could be due to selective face configurational processing issues. Alternatively, the lack of the inversion effect may rely on a feature-based processing (e.g., the position of the eyes) rather than a configural-based processing. In literature, however, it is widely accepted that face perception is holistic in nature, meaning that humans tend to process upright face stimuli as a whole (Taubert et al., 2011), while this holistic encoding becomes less efficient with inverted faces (Farah et al., 1995; Rossion, 2008, 2009). Looking at behavioral data from MCG, this pattern seems to be even more exacerbated considering higher accuracy rates and faster RTs for upright rather than inverted stimuli, thus supporting a holistic approach rather than a feature-based processing, in line with healthy participants. Moreover, comparing upright to inverted faces should offer the unique opportunity to control low-level visual features since the stimuli are equivalent in their low-level properties (Willenbockel et al., 2010). As a proof, no significant effects were found at the P100 level, which is known to reflect low-level visual processing stages. Nonetheless, recent evidence has suggested a larger role of facial features (i.e., eyes) for familiar face identity recognition, which actually occurs later in time affecting components like the N250 (Mohr et al., 2018).

Data from Experiment 2, where the emotional content was specifically manipulated, showed an enhanced N170 for upright fearful faces if compared to neutral faces, despite the impaired ability of a patient MCG to consciously recognize facial emotions. In line with previous research about negative emotions (e.g., Bennett and Hacker, 2005), this result reinforces the idea that emotional stimuli can be processed outside consciousness, given their high arousing content. This can represent a useful hint to develop specific rehabilitation interventions to alleviate or slow down the decline of face processing problems in patients with AD (Torres et al., 2015; Torres Mendonça De Melo Fádel et al., 2019) by taking advantage of the recruitment of spared subcortical centers needed to process emotional stimuli.

Data from experiment 3, where familiarity was manipulated, did not show any modulation of the N250. This finding could be accounted for her inability to access the face representation stored in visual memory, i.e., her inability to consciously recognize familiar faces. However, another possibility can hold true, i.e., a false negative effect. Indeed, in healthy participants, we did not find a clear modulation of the N250 in the most typical topography, i.e., at the posterior electrodes. This lack of evidence could be caused by the characteristics of the paradigm used. The task of the participants was to report the orientation of the presented face, thus familiarity not being relevant for the task. In this respect, it has been reported that the modulation of the N250 is more likely to occur when familiarity is task-relevant, i.e., it is explicitly requested by the task to report whether a face is familiar or not (Schweinberger et al., 2002; Tanaka et al., 2006; Webb et al., 2010). Less controversial is the finding related to the N400 component. Indeed, we found a clear modulation, both in healthy controls and, more importantly, in patient MCG. Specifically, the N400, at posterior electrodes, was enhanced in amplitude for upright familiar faces as compared to that elicited by unfamiliar faces. This result is extremely relevant to assess the nature of face recognition deficit of MCG as related to either the perceptual or the memory component of face processing. Indeed, since the N400 is considered a correlate of face post-perceptual processing specifically related to the pure semantic stage (Bentin and Deouell, 2000; Jemel et al., 2010; Olivares et al., 2015), its modulation in patient MCG indicates that long-term memory traces are still preserved in this patient. Importantly, as she cannot overtly recognize faces, this effect has to be considered implicit. Again, this effect is not only important to identify the nature of the deficit but is also of extreme relevance for rehabilitation protocols. Indeed, if, at least implicitly, memory traces are preserved and can be recruited, specific interventions with the use of personally relevant familiar faces could serve to ameliorate or slow down face processing problems in patients with AD.

Taken together, the proposed approach may provide useful insight into the nature of face processing problems in patients with AD. We believe that the significance of this study is two-fold, for both basic and clinical science. These data suggest that a deep investigation of a single case, coupled with the use of electrophysiology, could lead to scientific advances (Mazzi and Savazzi, 2019). Specifically, this approach has been helpful in disentangling the perceptual deficit from memory causes of the deficit, thus providing a valuable methodology to be used in future research. Certainly, to ascertain whether face processing difficulties can be ascribed (also) to the perceptual stage in the general population of patients with AD, a larger study involving a group of patients must be carried out as a next step. Importantly, it remains to be established whether the gnosic nature of face recognition deficits can be restricted to the subpopulation of patients with AD with posterior onset of the disease (e.g., Peña-Casanova et al., 2012; Weintraub et al., 2012), while for other patients, a memory nature could better explain the deficit. Both possibilities are at place, although it has recently been reported that tau deposition (a biomarker of AD) in the inferior temporal cortex, an important brain region for recognition of faces and objects in general, tends to be an early sign of AD (e.g., Cho et al., 2016; Johnson et al., 2016) and can be predicted in clinically normal elderly participants with cardiovascular disease (Rabin et al., 2019). This piece of evidence, together with the low rate of specific assessment in clinical practice of face processing deficits, or agnosia in general, clearly leading to an underestimation of the impact of visuoperceptual deficits in patients with AD, suggests the possibility that a perceptual deficit could be present in a considerable portion of patients with AD (Lavallée et al., 2016), at least at a mild stage of the illness.

In this respect, the use of electrophysiological markers in patients with AD (Rossini et al., 2020) has proved to be a valuable support to detect early biomarkers (Swanwick et al., 1999; Jackson and Snyder, 2008; Lai et al., 2010) capable to differentiating a mild AD from normal aging (Swanwick et al., 1996) and to reveal disease progression (Lai et al., 2010). Moreover, the data presented here suggest that electrophysiological biomarkers can be informative in the differential diagnosis among cognitive function deficits by revealing their neuropsychological etiology. These pieces of evidence, together with the suitability and easiness of the use of EEG in outpatient settings (Cecchi et al., 2015), strongly warrant the use of electrophysiological markers in the AD population (Babiloni et al., 2020).

In conclusion, face recognition has a crucial role in determining the quality of social interaction and the psychosocial well-being of a patient, which in turn play an essential role in dementia care (Donix et al., 2013). Therefore, it is our opinion that a better assessment of specific functions through electrophysiological markers could be of paramount importance in clinical practice to obtain a more reliable estimation of the incidence of these deficits in AD population and thus to improve the classification and management of patients. Most importantly, this approach could help to devise *ad hoc* rehabilitation protocols for face processing deficits (Hawley and Cherry, 2004), serving the important goal of reducing the loss of self-confidence and the rate of social withdrawal typically found in patients with AD as the illness progresses.

## Data Availability Statement

The datasets generated for this study can be found in online repositories. The names of the repository/repositories and accession number(s) can be found below: https://figshare.com/s/06414ec1d201866ec5fe.

## Ethics Statement

The studies involving human participants were reviewed and approved by Comitato etico indipendente per l'approvazione di studi e sperimentazioni cliniche of the Department of Neuroscience, Biomedicine and Movement, University of Verona. The patients/participants provided their written informed consent to participate in this study. Written informed consent was obtained from the individual(s) for the publication of any potentially identifiable images or data included in this article.

## Author Contributions

SS and GM contributed to the conception and design of the study. CM and SS programed the experiments and contributed to the data acquisition and organization of the database. LD contributed to patient's referral, supervised instrumental and biological testing, and provided the neurological diagnosis. GM contributed to the patient's neuropsychological assessment and data scoring and analysis. CM performed preprocessing and statistical analysis of behavioral and EEG data. CM, SS, and JS-L discussed data analyses and contributed to data organization. SS, CM, and GM wrote the first draft of the manuscript. All authors contributed to manuscript discussion, revision, reading, and finally approved the submitted version.

## Conflict of Interest

The authors declare that the research was conducted in the absence of any commercial or financial relationships that could be construed as a potential conflict of interest.
